# Government Actions and Their Relation to Resilience in Healthcare During the COVID-19 Pandemic in New South Wales, Australia and Ontario, Canada

**DOI:** 10.34172/ijhpm.2021.67

**Published:** 2021-07-06

**Authors:** Andrew Smaggus, Janet C. Long, Louise A. Ellis, Robyn Clay-Williams, Jeffrey Braithwaite

**Affiliations:** ^1^Queen’s University, Kingston, ON, Canada.; ^2^Australian Institute of Health Innovation, Macquarie University, Sydney, NSW, Australia.

**Keywords:** Resilience, Resilience in Healthcare, Complexity, New South Wales, Ontario

## Abstract

**Background:** Resilience, a system’s ability to maintain a desired level of performance when circumstances disturb its functioning, is an increasingly important concept in healthcare. However, empirical investigations of resilience in healthcare (RiH) remain uncommon, particularly those that examine how government actions contribute to the capacity for resilient performance in the healthcare setting. We sought to investigate how governmental actions during the coronavirus disease 2019 (COVID-19) pandemic related to the concept of resilience, how these actions contributed to the potential for resilient performance in healthcare, and what opportunities exist for governments to foster resilience within healthcare systems.

**Methods:** We conducted case studies of government actions pertaining to the COVID-19 pandemic in New South Wales, Australia and Ontario, Canada. Using media releases issued by each government between December 2019 and August 2020, we performed qualitative content analysis to identify themes relevant to the resilience potentials (anticipate, monitor, respond, learn) and RiH.

**Results:** Direct references to the term ‘resilience’ appeared in the media releases of both governments. However, these references focused on the reactive aspects of resilience. While actions that constitute the resilience potentials were evident, the media releases also revealed opportunities to enhance learning (eg, a need to capitalize on opportunities for double-loop learning and identify strategies appropriate for complex systems) and anticipating (eg, incorporating the concept of hedging into frameworks of RiH).

**Conclusion:** Though fostering RiH through government action remains a challenge, this study suggests opportunities to realize this goal. Articulating a proactive vision of resilience and recognizing the complex nature of current systems could enhance governments’ ability to coordinate resilient performance in healthcare. Reflection on how anticipation relates to resilience appears necessary at both the practical and conceptual levels to further develop the capacity for RiH.

## Background

**Key Messages**

** Implications for policy makers**

- This study addresses a gap in the resilient healthcare literature by investigating government actions and their relation to resilience in healthcare (RiH).
- While the technical concept of resilience was not directly invoked, the elements of resilience were evident in the actions of the governments of both New South Wales, Australia and Ontario, Canada during the coronavirus disease 2019 (COVID-19) pandemic.
- Embracing resilience as an organizing principle could help governments coordinate their preparation and responses to disruptive events.
- A focus on learning relevant to the nature of complex systems represents an opportunity to enhance resilience throughout healthcare.
- In addition to the ongoing development of relevant practices, fostering RiH will require ongoing evaluation and development of the theory and conceptual foundations of resilience (particularly concerning anticipation).

** Implications for the public**

Resilience is the ability of a system to maintain a desired level of performance when circumstances disturb its functioning. This concept has become an important contributor to healthcare quality and safety, however studies of resilience in real-world settings remain uncommon. This investigation of governmental actions in New South Wales, Australia and Ontario, Canada during the coronavirus disease 2019 (COVID-19) pandemic revealed opportunities to enhance resilience in healthcare (RiH) systems. Articulating a clear vision of resilience, capitalizing on opportunities for double-loop learning (eg, learning about the nature of complex systems), and developing the conceptual foundations of resilience (eg, adapting the concept of anticipation in a manner that better informs practice) are all opportunities to enhance resilience within healthcare. Governments that address these areas could enhance the ability of their healthcare systems to respond to challenges and unexpected events, which could ultimately improve the quality and safety of care provided to patients.

Resilience has emerged as a key concept in efforts to enhance the quality and safety of healthcare around the world. Based on ideas from ecology,^1^ resilience refers to a system’s ability to maintain a desired level of performance when circumstances disturb its functioning.^2-7^ This concept has contributed to advances in numerous fields in recent decades.^8,9^ The application of resilience to the healthcare setting has arisen from safety science (ie, Resilient Health Care, which examines the capacity of healthcare systems to maintain required levels of performance through adaptions before, during, and after challenges and disruptions)^4,10-12^ and disaster resilience (ie, health systems resilience, the application of resilience thinking in public health responses to major crises such as natural disasters and outbreaks of infectious diseases).^6,13-15^ With a common foundation in the principles of resilience, these applications are all united under the term “resilience in healthcare” (RiH).^16^ RiH is defined as the capacity to consistently deliver safe, high-quality healthcare through adaptations at multiple systems levels in the face of challenges and disruptions.^16^ This definition encompasses resilience-based efforts and analysis at all levels of healthcare.^16^

 RiH provides a number of concepts and strategies to address the challenge of maintaining safe, high-quality healthcare amidst expected and unexpected challenges and disruptions.^16^ It recognizes the adaptive capacity of a healthcare system as foundational to its ability to provide high-quality care.^7,11,16^ RiH also emphasizes the importance of learning from all events (not just those with adverse outcomes) and appreciating the differences between work-as-done (WAD) and work-as-imagined (WAI) (ie, understanding the significance of differences between the way frontline practitioners perform work versus the way those who design, evaluate, and regulate work believe it is performed).^4,7,10-12,16^ RiH also addresses the macro-level coordination of healthcare efforts in the face of large-scale crises (eg, a pandemic).^9,16^ This concern involves attention to issues of governance, workforce, and service delivery.^6,13,16^ At the foundation of RiH are the four resilience potentials (responding, monitoring, anticipating, learning), proposed by Hollnagel as the requirements a system must possess to be able to perform in a resilient manner.^16,17^ These potentials serve as a framework frequently employed in the analysis of RiH.^6,11,15^

 These concepts illustrate the close links between RiH and the discipline of complexity.^7,16^ RiH recognizes healthcare as a complex, socio-technical system and provides approaches that address the unique challenges of coordinating and maintaining safe, high-quality performance within such systems.^4,5,10,18^ This differentiates the resilience-based approaches of RiH from conventional approaches to healthcare quality and safety, which often struggle to address the challenges of complex systems.^10,19,20^

 Despite its conceptual appeal, the challenge of translating the principles of resilience into concrete improvements remains. To date, the RiH literature has focused predominantly on theory rather than empirical studies.^2,6^ Furthermore, much of the existing empirical research focuses on clinical microsystems (eg, emergency departments, intensive care units, or surgical settings within a single hospital).^2^ There is a particular lack of empirical investigations of macro-level activities (ie, actions of governments and regulatory agencies) and their relation to the resilience of healthcare systems.^11,21-23^ While the resilience literature on public health responses to natural disasters and outbreaks of infectious disease addresses macro-level concerns, a recent review found that these studies often focus on service delivery, leaving issues such as governance underexplored.^6^ As a result, a need exists for investigations of the relationship between government actions and the capacity for resilient performance in healthcare.

 Developing an understanding of how governments foster (or compromise) the capacity for resilience within healthcare is a crucial task. As healthcare systems at the micro, meso, and macro levels grow more complex, we require organizing principles that address the challenges of complexity at all levels. Though work at the level of clinical microsystems is vital to resilient performance, an excessive focus on frontline work risks neglecting the importance of action at the meso and macro levels. Similarly, actions at the meso and macro levels can prove counterproductive if they do not align with the goals, tasks, and challenges at the frontlines. At a time when clinician wellness and burnout are significant concerns,^24,25^ healthcare needs frameworks that distribute the responsibility for creating safe, high-quality care appropriately across all levels and avoid downloading excessive demands onto the shoulders of frontline workers. A greater understanding of how to foster resilience at the macro-level (eg, through government action) could provide necessary slack^26,27^ to alleviate some of the pressures experienced at the frontlines during unexpected disruptions.

 The coronavirus disease 2019 (COVID-19) pandemic represents an archetypal disruption to healthcare systems around the world. Reports of a novel coronavirus (eventually named SARS-CoV-2 [severe acute respiratory syndrome coronavirus 2]; COVID-19 refers to the disease caused by the SARS-CoV-2 virus)^28^ first emerged from China in late December 2019.^29^ Within a few months, SARS-CoV-2 had spread across the world, prompting the World Health Organization (WHO) to declare a pandemic on March 11, 2020.^29^ The virus proved highly transmissible and capable of causing severe disease (especially in those over age 60).^30-32^ Through 2020 and 2021, the pandemic has presented challenges that healthcare systems must address to maintain a desired level of performance.^33,34^ These challenges have required governments that administer healthcare systems to adapt in order to absorb the shock of the virus and coordinate the capacities needed to deal with its impact.^35-37^ Amidst these challenges and unfortunate circumstances, the pandemic provides a real-world opportunity to investigate resilience at a macro-level and how governments contribute to resilient performance in healthcare.

 To investigate how government action has contributed to resilient performance during the COVID-19 pandemic, we conducted a qualitative evaluation of the actions taken by two governments that directly oversee their healthcare systems (the state government of NSW, Australia and the provincial government of Ontario, Canada) during the initial months of the pandemic. We used official public communications and media releases issued by these governments and their respective departments and ministries to investigate (*i*) how do the executive and legislative actions of the government relate to the technical concept of resilience? (*ii*) how do these actions contribute to the capacity for resilient performance within healthcare? and (*iii*) what opportunities exist to foster resilient performance within healthcare through government action?

###  Conceptual Framework

 This study used Hollnagel’s resilience potentials as a framework for evaluating resilience. Hollnagel proposed that four potentials – responding, monitoring, anticipating, and learning – are fundamental requirements for resilient performance in any domain. Together, these potentials constitute the systemic property of resilience. Though described individually, they are interdependent and meant to be analyzed in concert.^17^

 The potential to respond refers to the actions taken following anticipated or unanticipated changes, disturbances, or opportunities. Responding may involve activating previously prepared actions (eg, activating a pandemic plan), altering existing actions (eg, re-organizing services to create healthcare capacity), or creating new actions (eg, introducing new requirements for indoor ventilation). In addition to identifying how to respond, identifying when to respond is integral to this potential.^17^

 The potential to monitor refers to the act of identifying events that signal the need to respond. It involves observing and measuring both a system’s performance (eg, tracking case counts and deaths) and the elements of the environment that can affect performance (eg, availability of intensive care beds and ventilators). Both the internal and external environments of a system are relevant to monitoring.^17^

 The potential to anticipate refers to the capacity to create a model of the future that includes the possible disruptions (eg, new variants of concern), demands (eg, increased need for intensive care beds), and opportunities (eg, the opportunity to utilize mRNA vaccines) a system may face. Hollnagel differentiated anticipation from monitoring by stressing the role of imagination in anticipating. Where monitoring consists of observing that which is occurring, anticipating involves imagining the possible ways in which the future could unfold. Hollnagel also specified three modes of anticipation that contribute to this potential. The first is the mechanistic mode of anticipation that views the future largely as a repetition of the past (eg, if circumstances of a pandemic match those found in a past pandemic, we can expect events will unfold similarly). The second is the probabilistic mode that uses past trends and events to extrapolate a vision of the future (eg, using quantitative modelling to predict how case numbers will evolve). The third mode uses historical and present circumstances to infer novel future scenarios based on theories of how events arise. Hollnagel labelled this third mode realistic anticipation as it acknowledges that the future can differ fundamentally from the past and pose entirely new challenges (ie, challenges that differ qualitatively from the past, not simply quantitatively).^17^

 The potential to learn refers to a system’s ability to develop new knowledge, skills, and capabilities based on experience. Both single loop (ie, modifying action based on experience, such as introducing fines to prevent individuals from going into work while experiencing symptoms of COVID-19) and double loop (ie, using experience to evaluate and modify the assumptions, goals, and objectives that underlie action, such as recognizing the socio-economic conditions that necessitate individuals working while symptomatic) learning are necessary for resilient performance.^17,38^

 In considering the actions that constituted each of the resilience potentials at different levels, we utilized a recent framework for researching resilient performance that organizes activities at various levels of healthcare according to the potentials they address.^11^ We also considered the descriptions of the activities of governance, leadership, financing, information management, and workforce management located in health systems resilience literature, and how these actions relate to the resilience potentials based on the framework.^13^ We believed that a government’s contributions to resilience and resilient performance occurred through actions that address resilience potentials.

 Effects on resilience also occur through the manner in which governments leverage their actions. Woods described four conceptions of resilience (resilience as rebound, resilience as robustness, resilience as graceful extensibility, and resilience as sustained adaptability)^3^ that we regarded as different perspectives a government could take to leverage their efforts relevant to resilience.

 Resilience as rebound sees resilience as a system’s ability to recover from disruptions and restore its equilibrium. Resilience as robustness sees resilience as a system’s capacity to absorb the effects of disruptions (eg, through optimizing worst-case scenarios). Resilience as graceful extensibility refers to a system’s ability to stretch its capacity in the face of a disruption, ie, to find new ways to address challenges when operating at or near the boundaries of its capabilities. Woods contrasts graceful extensibility with brittleness, ie, the rapid deterioration of performance when operating at or near a system’s boundaries. Finally, resilience as sustained adaptability locates resilience in the structure, organization, and principles of a system that establish an ongoing capacity for ongoing adaptability as the system faces new disruptions and challenges.^3^

 Woods noted the importance of specifying the precise conception one is using when discussing efforts to develop or study resilience. The four conceptions are often conflated, yet they have different ramifications for efforts to develop resilience. We considered the conceptions of resilience as guiding principles that influence actions taken and how they are leveraged. As these actions constitute the resilience potentials, different conceptions lead to differences in the relative amount of attention given to each resilience potential. For example, viewing resilience as the ability of a hospital or ward to rebound following a disturbance may lead to reactive efforts focused on recovery, whereas viewing resilience as graceful extensibility may prove more conducive to proactive efforts to minimize brittleness.^3^ The former could lead to an excessive focus on responding, while the latter could lead to a more productive balance of the four potentials.

 Furthermore, Woods noted that the more proactive concepts of graceful extensibility and sustained adaptability have contributed more to the advancement of theory and development of practices than the more reactive concepts of rebound and robustness.^3^ Therefore, it is key to recognize the importance of the proactive aspects and avoid conceptualizing resilience as simply reactive. Though Woods focused primarily on the study and investigation of resilience, we considered his descriptions applicable to practical efforts to foster resilience.

 A summary of the resilience potentials, conceptions of resilience, and the relationship between them is presented in Supplementary file 1.

## Methods

 We conducted descriptive case studies of the official communications issued by the governments of (*i*) NSW, Australia and (*ii*) Ontario, Canada regarding actions taken during the COVID-19 pandemic. Case study methods were used because of their potential to provide a detailed picture of complex phenomena (eg, resilience) and their utility in settings where researchers have limited control over events and behaviours (eg, during a pandemic).^39,40^ Through analysis and comparison of government actions in each jurisdiction, we aimed to identify high-level themes regarding resilience relevant to healthcare (rather than a direct comparison of outcomes).

###  Context

 We selected NSW and Ontario as the jurisdictions to study for several reasons. First, both jurisdictions had histories that could produce unique approaches relevant to resilience. Ontario experienced an outbreak of the severe acquired respiratory syndrome (SARS) in 2003, and a subsequent commission was critical of actions taken by leaders in government and healthcare during the outbreak.^41^ With memories of the SARS outbreak persisting,^42-44^ we believed that experience would impact Ontario’s efforts to maintain performance during the COVID-19 pandemic. In NSW, the government had previously demonstrated a desire to foster resilience at a systemic level.^45^ Furthermore, the catastrophic 2019-20 bushfires that preceded COVID-19 were felt to provide learnings and reflection that could promote resilient behaviour in response to a new disturbance.^46,47^

 The second reason for selecting these jurisdictions was the familiarity of the research team with the local contexts in these areas, which we believed would provide advantages during the data analysis. In the preceding years, Ontario had launched efforts to restructure its healthcare system to enhance the integration of services and address issues of capacity and sustainability.^48-50^ These efforts involved changes to healthcare infrastructure (eg, the creation of “Ontario Health Teams”) intended to provide patients with more direct access to their providers and assist them in navigating transitions (eg, from hospital to home).^51^ These changes were intended to enhance the care patients receive and to address capacity issues facing hospitals (ie, to end “hallway medicine”).^52^ Implementation of these reforms was in progress at the time the COVID-19 pandemic appeared.

 Comparable reforms in the NSW healthcare system began in 2011, with changes that shifted greater authority to local health districts. Much like in Ontario, these reforms were implemented to enhance patient access to services and address growing issues with hospital capacity.^53^ A subsequent audit of these reforms was largely positive but identified ongoing issues that continue to challenge the healthcare system (eg, clinician engagement, residual ambiguity in responsibilities of local health districts).^54^

 In addition to facing similar challenges, NSW and Ontario share similar demographics (eg, both are the most populous regions in their country, with predominantly English-speaking populations and similar levels of economic prosperity) and health systems (eg, both systems are largely publicly funded; both governments are directly responsible for administering their acute care systems, with regulatory oversight and funding provided by their respective federal governments).^55^ These similarities minimized the number of confounding factors to the extent possible and allowed a focus on the acute efforts regarding COVID-19. Table 1 provides further details regarding relevant demographics.

**Table 1:** Demographics and healthcare metrics of NSW, Australia and Ontario, Canada

**Demographic**	**NSW**	**Ontario**
Population	8 164 128^56^	14 733 119^57^
GSP (NSW) or GDP (ONT) (2019, millions of US dollars)	$426 520^58^	$735 211^59^
GSP or GDP per capita (2019, US dollars)	$53 062^58^	$50 548^57,59^
Healthcare spending per capita (2019, US dollars)	$5162^60^	$5732^61^
Acute care beds per capita (per 1000 population)	3.9^62^	1.7^63^
Healthcare Administration	Largely publicly funded; state is responsible for acute care (federal government retains authority for primary care)^55^	Largely publicly funded; province is responsible for acute care and primary care^55^
Historical events relevant to COVID-19 pandemic	2009 H1N1 influenza pandemic, 2020 Australian Bush Fires	2003 SARS outbreak, 2009 H1N1 influenza pandemic

Abbreviations: NSW, New South Wales; ONT, Ontario; GSP, gross state product; GDP, gross domestic product; COVID-19, coronavirus disease 2019; SARS, severe acute respiratory syndrome. Note: Dollar amounts, originally reported in local currencies, were converted to US dollars using purchasing power parities (PPP) for the appropriate year as reported by the Organisation for Economic Co-Operation and Development (OECD).^64^

 Regarding the events of the pandemic, both regions reported their first case of COVID-19 on January 25, 2020.^65,66^ They also reported their hundredth cases at similar times (March 13, 2020 in Ontario, March 14, 2020 in NSW).^67,68^ The first death from COVID-19 in NSW occurred on March 3, 2020, while Ontario’s first death from COVID-19 was reported on March 17, 2020.^69,70^ While these initial events unfolded similarly, Ontario subsequently saw a more rapid increase in cases and deaths.^67,71,72^ Graphs of the cumulative number of cases and deaths during the study period are available in Supplementary file 2.

###  Data Sources and Data Collection

 The primary data for this study were obtained from media releases relevant to the COVID-19 pandemic issued by the governments of NSW and Ontario. Media releases from the NSW government and its departments were collected from the respective governmental websites (accessed via https://www.service.nsw.gov.au/nswgovdirectory/departments). Media releases from the Ontario government were obtained from their online newsroom (https://news.ontario.ca/newsroom/en). The structures of these governments and their departments and ministries are presented in Supplementary file 3.

 Media releases relevant to the COVID-19 pandemic were identified by searching the above websites for the terms “COVID-19,” “COVID,” “coronavirus,” and “pandemic.” One researcher (AS) collected media releases containing any of these terms dating from December 2019, when reports of a novel coronavirus first emerged,^29,73^ until August 31, 2020. The chosen end date coincided with the return of children to schools in Ontario^74^ and the end of winter in NSW. As these changes had the potential to represent a new phase of the pandemic and pandemic response, we decided to stop data collection on that date.

 All relevant documents were imported into NVivo qualitative analysis software, version 12.6.0 (QSR International, Doncaster, Victoria, Australia) for analysis.

###  Data Analysis

 We performed qualitative content analysis^75,76^ on the content of the media releases. One researcher (AS) developed a deductive coding framework based on key concepts relevant to resilience (with a focus on Hollnagel’s descriptions of the resilience potentials).^8,11,17^ The same researcher (AS) coded all of the media releases line-by-line. Three researchers (JL, RCW, LE) reviewed the coding framework and each independently coded a sample of media releases. Codes were compared and discrepancies resolved through discussion, leading to refinement of the coding framework. On a second pass, the initial researcher (AS) reviewed and refined codes based on the revised framework. In the subsequent inductive stage, codes were combined by one researcher (AS) to derive themes relevant to healthcare. These themes were developed and refined in multiple group meetings among the researchers. Themes were checked for consistency by three researchers (JL, RCW, LE).

## Results

 A total of 1084 media releases relevant to COVID-19 were identified between December 1, 2019 and August 31, 2020. NSW issued 670 media releases related to COVID-19. Ontario issued 414 media releases related to COVID-19 during the period of study. The number of media releases issued per month is presented in Supplementary file 4.

###  Direct References to Resilience

 Media releases from both governments contained several direct references to resilience. While these references largely used the term resilience to describe a positive attribute of individuals, communities, and industries, a precise definition of resilience was not provided.

*“The NSW community has shown extraordinary resilience in the face of many disasters – bushfires, drought, flood and now the COVID-19 pandemic,” [NSW Premier Gladys] Berejiklian said. *(NSW Office of the Premier & Deputy Premier 2020 04 06).

*“The resiliency of everyday people, supported by coordinated action from all levels of government, is the bedrock of Ontario and Canada”* (Ontario Statement 2020 03 13).

 A significant direct reference to resilience occurred in NSW with the establishment of a new government agency (Resilience NSW) dedicated to disaster preparation and recovery. Though the term was not precisely defined, the mission of the agency suggested resilience as the ability to prepare, coordinate activities, and rebound from disturbances.

*“Resilience NSW will lead the whole-of-government prevention, preparedness and recovery effort. It will oversee and coordinate emergency management policy, service delivery and all aspects of disaster recovery at a state, national and international level,” [Police] Commissioner Fitzsimmons said. “There was never a more important time to make sure that communities devastated by drought, bushfires and now COVID-19 are getting the help they need to rebuild and recover”* (NSW Premier-Deputy Premier 2020 04 06).

 A similar initiative was referenced in the Ontario media releases, though this program was primarily developed by the federal government of Canada. Again, the term resilience was not defined explicitly. However, these releases suggested the concept of recovery (primarily through financial support).

*“The COVID-19 Resilience Stream will help other orders of governments whose finances have been significantly impacted by the pandemic by increasing the federal cost share for public infrastructure projects”* (Ontario News Release 2020 08 27).

*“…a new stream has been added to the over $33-billion Investing in Canada Infrastructure Program to help fund pandemic-resilient infrastructure”* (Ontario News Release 2020 08 28).

###  Resilience Potentials

 Actions related to the resilience potentials were frequently described in the media releases from both NSW and Ontario. Unlike the direct references to resilience, references relevant to the resilience potentials frequently addressed healthcare and issues of public health.

###  Responding

 Responding was the resilience potential most commonly identified within the media releases. Representative quotes pertaining to the potential to respond are presented in Table 2.

**Table 2:** Representative Quotes Pertaining to the Potential to Respond in Media Releases Issued by the NSW Government and the Government of Ontario

**Potential to Respond**	
Imposing New Restrictions	
*“Today, the Government of Ontario announced that it is taking decisive action by making an order declaring an emergency under s 7.0.1 (1) the Emergency Management and Civil Protection Act. In doing so, Ontario is using every power possible to continue to protect the health and safety of all individuals and families” *(Ontario News Release 2020 03 17).	
*“The NSW Government has taken significant new steps to increase restrictions across the state – triggering the next level of enforcement necessary to fight COVID-19. Following the decisions made by National Cabinet, NSW Premier Gladys Berejiklian confirmed the shutdown to protect NSW citizens” *(NSW Premier-Deputy Premier 2020 02 23).	
Relaxing Pre-Pandemic Restrictions	
*“Families will have more access to influenza vaccinations with the NSW Government lowering the age pharmacists can administer flu jabs to children from 16 years to 10 years. Health Minister Brad Hazzard said giving families more options to protect their children against flu is sensible, with the likely convergence of a COVID-19 pandemic with winter flu”* (NSW Health Minister 2020 03 16).	
*“To ensure that anyone in need of care can receive it, Ontario is waiving the three-month waiting period for OHIP coverage”* (Ontario News Release 2020 03 20). *“NSW pharmacists now have extra powers enabling them to dispense medicines without a prescription and can now stay open 24/7, as the State fights COVID-19. Minister for Health and Medical Research Brad Hazzard said a special authority has been granted to community pharmacists to assist people who can’t access their GP” *(NSW Health Minister 2020 03 31).	
Responding to New Challenges & Opportunities	
*“…we must now bolster our mental health system to ensure it is able to dynamically respond to future pressures…In NSW, we have already invested an extra $73 million in mental health supports to improve the wellbeing of the whole community” *(NSW Minister of Mental Health 2020 05 14).	
*“Starting today, the Ontario government is expanding virtual mental health services to help thousands of Ontarians experiencing anxiety and depression, including frontline healthcare workers, during the COVID-19 outbreak” *(Ontario News Release 2020 05 05).	

Abbreviations: NSW, New South Wales; COVID-19, coronavirus disease 2019; OHIP, Ontario Health Insurance Plan; GP, general practitioner.

 The responses to COVID-19 by both governments involved imposing restrictions (through new regulations and orders). At early stages of the pandemic, both NSW and Ontario imposed emergency orders that placed restrictions on activities of citizens (eg, cancelling public gatherings, closing non-essential services). These orders were deemed necessary to protect the public from the dangers of COVID-19 and limit its spread.

 Both governments also relaxed numerous pre-pandemic regulations and restrictions (eg, waiving the waiting period for access to health insurance) given new concerns for public safety. They also responded to new challenges and opportunities that the pandemic presented. NSW and Ontario both introduced programs to address mental health challenges.

###  Monitoring

 Government communications that addressed monitoring focused on epidemiological and public health metrics in both NSW and Ontario. Both governments stressed the importance of testing and contact tracing once cases began to emerge in their jurisdictions. As the pandemic evolved, references to delays in non-COVID care became more prominent. Representative quotes pertaining to the potential to monitor are presented in Table 3.

**Table 3:** Representative Quotes Pertaining to the Potential to Monitor in Media Releases Issued by the NSW Government and the Government of Ontario

**Potential to Monitor**	
Public Health Monitoring	
*“In line with national COVID-19 control guidelines, NSW Health has increased testing in areas which may be at elevated risk of community transmission. We are encouraging people in these areas who have symptoms including fever and/or flu-like symptoms such as cough, sore throat or shortness of breath to be tested” *(NSW Health Statement 2020 04 07).	
*“Working with Public Health Ontario and Ontario Health, the province is working to enhance laboratory testing capacity across the province to rapidly mobilize, monitor and coordinate COVID-19 testing” *(Ontario News Release 2020 03 12).	
Monitoring Delays in Non-COVID-19 Care	
*“Surgery lists are being closely monitored, and any patient whose condition changes or deteriorates should speak to their treating clinician” *(NSW Health Minister 2020 06 16).	

Abbreviations: NSW, New South Wales; COVID-19, coronavirus disease 2019.

###  Anticipating

 Anticipation of greater demand for healthcare services was a major theme evident in the media releases. This projection for greater demand (higher numbers of patients, higher numbers of critically ill patients) was tied to responses such as stockpiling personal protective equipment and procuring ventilators.

 Projections based on modelling used data to extrapolate future scenarios. These modelling projections were more prominent in Ontario’s media releases. Representative quotes pertaining to the potential to anticipate are presented in Table 4.

**Table 4:** Representative Quotes Pertaining to the Potential to Anticipate in Media Releases Issued by the NSW Government and the Government of Ontario

**Potential to Anticipate**	
Healthcare Capacity	
*“We have been prudently planning and regularly reviewing everything from emergency department and intensive care capacity, staff capacity and training and supplies of critical medical equipment to streamlining how we manage patients with acute respiratory illness … the situation for our hospitals could change quickly so we’re asking everyone to plan now” *(NSW Health Minister 2020 02 27).	
*“…the Ontario government has significantly expanded hospital capacity in preparation for any COVID-19 outbreak scenario. The province has added 1,035 acute care beds and 1,492 critical care beds and taken steps to ensure hospitals have the staff available to care for a sudden surge in patients” *(Ontario News Release 2020 04 16).	
Projections from Modeling	
*Today, the Ontario government released extensive COVID-19 modelling, revealing several scenarios that project the potential number of cases and deaths. In doing so, the province is providing the public with full transparency about the consequences should everyone but essential workers fail to stay home and practice physical distancing” *(Ontario News Release 2020 04 03).	

Abbreviations: NSW, New South Wales; COVID-19, coronavirus disease 2019.

###  Learning

 Formal learning efforts described in the media releases involved inquiries into problems that arose during the pandemic. The inquiry regarding the outbreak associated with the *Ruby Princess* cruise ship in NSW and the investigation of the COVID-19 response in long-term care homes in Ontario were the main examples of formal learning pursuits in each jurisdiction.

 Government communications in NSW occasionally referenced lessons learned from reviews of prior pandemics and outbreaks. Similar references were rare in Ontario’s media releases. Despite the province’s extensive experience with the SARS outbreak in 2003, references to SARS were limited to a mention that changes to staff deployment were also pursued during the SARS outbreak in a news release on March 28th and another news release on July 29th that stated an independent commission (like that pursued regarding the effects of COVID-19 in long-term care) was also conducted following SARS (see Supplementary file 5 for relevant quotes from these news releases).

 Lessons learned from experiences in other countries and from earlier local experiences appeared in the government communications. These lessons impacted subsequent actions (single-loop learning). Though more difficult to identify, examples of lessons that affected fundamental goals, objectives, and underlying assumptions (double-loop learning) also manifested, often related to beliefs and assumptions about the virus (eg, its transmissibility). Representative quotes pertaining to the potential to anticipate are presented in Table 5.

**Table 5:** Representative Quotes Pertaining to the Potential to Learn in Media Releases Issued by the NSW Government and the Government of Ontario

**Potential to Learn**	
Formal Inquiries & Investigations	
*“A special commission of inquiry will be established to investigate the events surrounding the Ruby Princess cruise ship” *(NSW Premier-Deputy Premier 2020 04 15).	
*“The Ontario government launched an independent commission into COVID-19 and long-term care. Three commissioners will investigate how COVID-19 spread within long-term care homes, how residents, staff, and families were impacted, and the adequacy of measures taken by the province and other parties to prevent, isolate and contain the virus. The commission will also provide the government with guidance on how to better protect long-term care home residents and staff from any future outbreaks” *(Ontario News Release 2020 07 29).	
Lessons Learned from Australian Bushfires	
*"The past few months have been incredibly challenging both for school communities in bushfire and now flood impacted areas," [Minister for Education Sarah] Mitchell said…."We have learnt you can never over-communicate in a crisis…. During the fires last year and floods this year, schools worked incredibly hard to ensure everyone was informed”* (NSW Dept. of Education 2020 03 02).	
Lessons Learned from Previous Outbreaks	
*“The NSW Health response to COVID-19 is part of an existing framework for managing emerging infectious diseases, including pandemic influenza, SARS and MERS in the past, which has been refined over many years”* (NSW Health Release 2020 02 13). *“Health Minister Brad Hazzard and Chief Health Officer Dr Kerry Chant said NSW Health has planned extensively for a pandemic and further strengthened its response since the SARS, MERS and H1N1 ‘swine flu’ threats, but everyone plays a role in prevention”* (NSW Health Minister 2020 02 27).	
Lessons Learned During the Pandemic	
*“…the emergence of community spread of COVID-19 in multiple countries outside mainland China demonstrates how quickly the virus can pass from person to person and, because it can present as mild disease, how preventing its spread can be challenging” *(NSW Health Minister 2020 02 27).	
*“Virtual healthcare has proven to be particularly effective for follow up and secondary appointments, after a patient’s initial diagnosis at a face-to-face consultation” *(NSW Premier-Deputy Premier 2020 07 15).	
*“NSW Health has recently started a research program to test sewage for traces of COVID-19 across the state … Initial samples collected at the Perisher sewage treatment plant on Wednesday 22 July 2020 returned a positive result for the presence of COVID-19” *(NSW Health Statement 2020 07 30).	

Abbreviations: NSW, New South Wales; COVID-19, coronavirus disease 2019.

###  Additional Themes

 In the early stages of the pandemic, Ontario often cited adherence to protocols as a reason for public confidence in the response to COVID-19. Quotes relevant to adherence repeatedly appeared in Ontario news releases between February 23rd and March 8th.

*“All Protocols Followed and Risk to Ontarians Remains Low” *(Ontario News Release 2020 02 23).

*“As per established infection prevention and control protocols, the patient was cared for at the hospital using all appropriate precautions, including being isolated as she was tested for COVID-19” *(Ontario News Release 2020 02 26).

 Some media releases contained information that would appear dubious at later stages of the pandemic. The contrast between the earlier statements and those that appeared later arose abruptly with limited explanation of how these lessons were learned.

*“This virus does not appear to spread easily between people”* (NSW Health Release 2020 01 21).

*“Face masks are not recommended for the general public unless you are unwell and masks should be saved for people to use when they are sick” *(NSW Health Minister 2020 02 27).

## Discussion

 This study of government-issued media releases pertaining to the COVID-19 pandemic in NSW, Australia and Ontario, Canada suggests that actions that constitute resilient performance are woven throughout government activities. However, the manner in which governments discuss these actions suggests that opportunities exist to (*i*) articulate a specific, proactive vision of resilience that can aid the coordination of activities, and (*ii*) better develop the potentials of learning and anticipating.

###  Articulating a Proactive Vision of Resilience

 While both governments made direct references to resilience in their media releases, neither specified a precise meaning for resilience. This could represent an opportunity for governments to articulate a common purpose that facilitates coordination of activities and enhances a healthcare system’s ability to address unexpected disruptions. A clear statement that defines resilience could be a potent expression of purpose that aligns efforts across all levels of the system. It could also help to develop a discourse on resilience that is not simply about reacting and recovering, but also about proactively establishing processes that build adaptive capacity on an ongoing basis.

 Without a clear expression of what resilience represents, the term may serve a rhetorical purpose rather than a practical, organizing purpose. Though appeals to resilience make governmental efforts sound positive, they are unlikely to provide a shared purpose or common goal without a clear meaning attached to the term. Acknowledging the technical concept of resilience as a systemic property worthy of attention,^8^ developing an understanding of that property (and how our actions impact it), and communicating that understanding to the public could create a clear goal that serves as an effective organizing principle.

 Furthermore, clarifying the meaning of resilience allows an opportunity to introduce its more proactive aspects to public discourse. As Woods mentions, the yield of the reactive resilience conceptions of rebound and robustness has proven low when it comes to stimulating new theories and practices relevant to resilience.^3^ The more proactive resilience conceptions of graceful extensibility and sustained adaptability may have greater potential to foster resilient performance. However, they are also less intuitive and awareness of them throughout society is likely low. Without clear descriptions of its proactive aspects, the use of the term resilience may be interpreted in the more generic, reactive sense and lose much of its potential utility.

 Evidence suggesting the preponderance of the reactive conceptions appears in reports from both NSW and Ontario. When announcing efforts to establish a government agency (NSW) or project (Ontario) to foster resilience, the media releases evoked ideas of recovery, rebound, and robustness. This is unfortunate as opportunities existed to highlight the importance of graceful extensibility and sustained adaptability. Efforts to stretch the capacity of the healthcare system to avoid deteriorations in performance (eg, through the proactive redeployment of staff to areas in need) were not linked to these references to resilience despite representing examples of what graceful extensibility could look like amidst the COVID-19 pandemic. Highlighting the importance of these actions and explicitly bringing them into discussions of resilience could help to expand the discourse of resilience within healthcare beyond rebound and recovery, and stimulate more productive discussions on how to address the proactive aspects of resilience.

 Articulating a specific concept of resilience could begin by making these links between existing actions and the more proactive forms of resilience explicit. Establishing these links could facilitate planning on how to re-organize resources when disruptions occur. It could also focus our learning on how to identify the boundaries of our systems, how to maintain capacity as the boundaries are approached, and what redistributions of resources facilitate the ability to gracefully extend our capacities in the face of challenges.

 At the same time, articulating a definition and vision of resilience may be necessary but not sufficient to produce resilient performance.^23^ Opportunities to develop the actions and practices that constitute the resilient potentials provide additional insights regarding this challenge.

###  Complexity and Opportunities for Double-Loop Learning

 Double-loop learning refers to the use of experience to re-evaluate and modify the assumptions, goals, and objectives that underlie our actions.^38^ In the media releases, some evidence of double-loop learning surfaced (eg, re-evaluations of assumptions about the transmissibility of the virus and the measures needed to limit transmission). However, there were important assumptions about healthcare systems that did not appear to receive re-evaluation. Specifically, re-evaluations of assumptions about the complexity of our systems were conspicuous by their absence. Instead, strategies more appropriate for simple, tractable systems persisted throughout the pandemic response.

 Understanding the differences between simple and complex systems is key to recognizing the importance of resilience. A complex system has a high degree of interconnectivity between elements, and it is largely the interconnections and interdependencies that determine the properties of the system. As a result, complex systems demonstrate emergent behaviours, self-organization, and other properties that distinguish them from simple, tractable systems.^77-79^ Given these different properties, actions that may be effective in simple systems (eg, developing protocols that specify responses and ensuring adherence to those protocols) can prove ineffective in complex systems.^18,80^ Despite the growing appreciation for the principles of complexity in many fields,^81,82^ in the current study it was difficult to identify communications that acknowledged the unique properties and challenges that complex, socio-technical systems present.

 This observation is particularly relevant to Ontario. The province’s media releases from the early months of the pandemic repeatedly mentioned adherence to protocols at the frontlines and presented that adherence as a reason for the public to have confidence in the government’s responses. These statements suggest a worldview informed by simple, tractable systems. They indicate an overestimation of the utility of prescriptive measures in the setting of incomplete knowledge, and limited awareness of the notions of WAI, WAD, and the importance of addressing the gap between them for system performance (eg, the importance of understanding why asymptomatic individuals may not observe quarantine requirements and continue to work following close contact with a person known to have COVID-19).

 This finding suggests the importance of capitalizing on opportunities for double-loop learning regarding complexity, ie, opportunities to evaluate assumptions about the nature of healthcare systems, how they operate, and how to approach improving them. However, an examination of Ontario’s previous response to SARS suggests these opportunities can go unfulfilled. In 2006, the province’s SARS Commission issued a report containing criticisms of the response to the outbreak and recommendations for the future. The report highlighted the importance of precaution, listening to frontline workers to understand WAD, and avoiding dogmatic thinking when facing novel threats. Though complexity and resilience are not explicitly mentioned in the full report, these suggestions provide guidance relevant to dealing with complex systems and align with many of the concepts that have subsequently emerged in discussions of RiH.^83^ In retrospect, the SARS Commission report provided an opportunity to evaluate the basis for these recommendations, what they suggest about the underlying nature of our systems, and how this understanding can be applied when responding to new threats to population health and safety. However, the COVID-19 media releases suggest that opportunity for double-loop learning was not fully realized.

 More specifically, the early focus on the importance of adherence at the frontlines appears more appropriate for a simple system dealing with a well-understood threat than for a complex system dealing with a novel threat. In the latter situation, “all protocols followed” is not necessarily a reason for confidence. Though structure is needed in responses, an excessive focus on adherence may make a system more brittle when knowledge about a threat is still evolving. Furthermore, an excessive focus on adherence could induce blind spots at the macro-level of a system if there is a failure to recognize and learn from the improvisations needed at the micro-levels to maintain performance in the face of a novel challenge (ie, a failure to appreciate the gap between WAD and WAI). Governments must appreciate this gap if they are to contribute positively to the capacity for resilience in the face of a novel, evolving disruption.

 To the credit of the province, the focus on adherence and the significance attributed to it disappeared from the media releases by mid-March. This change could reflect a lesson that strict adherence to protocols at the frontlines was not as crucial as the province initially believed. It is also necessary to acknowledge the potential impact of hindsight bias in this discussion. Nonetheless, the challenge of capitalizing on these opportunities for double-loop learning about the complex nature of healthcare systems in the wake of a disturbance (and before a subsequent one) appears key to catalyzing the development of resilience.

 Meeting this challenge will require a multi-faceted strategy. Elements that will likely serve this goal include attention to processes of organizational learning,^84^ leadership that fosters a vibrant discourse in which novel ideas and strategies circulate in the public consciousness,^85^ and strategies to incorporate concepts of complexity into educational efforts and training programs (eg, to increase awareness of concepts such as non-linearity when dealing with complex socio-technical systems).^18^ While challenging, embedding lessons regarding complexity and complex systems into our learning and educational efforts could have significant positive implications for our responses to subsequent pandemics and disruptions.

###  The Challenge of Anticipation

 The anticipation evident in the media releases from both NSW and Ontario largely resembled the mechanistic and probabilistic modes of anticipation described by Hollnagel.^17^ Realistic anticipation was difficult to identify. As a result, anticipating during the early stages of the COVID-19 pandemic often resembled an exercise of preparing for more of the past (eg, more patients, more critically ill patients, more demand on the healthcare system) rather than imagining novel challenges looming in the future. In other words, we may be capable of anticipating a future that differs *quantitatively* from the past, but not necessarily one that differs *qualitatively* from the past.

 This finding regarding anticipation aligns with another recent finding that the practices related to resilience described in the literature often differ from the theory and conceptual foundations of resilience.^6^ While this draws attention to the need to develop practices that foster resilience, it also presents the field of resilience with a reflective, “looking-glass” moment. These findings suggest, to use the vocabulary of resilience, a gap between “resilience-as-imagined” and “resilience-as-done.” As with gaps between WAI and WAD, this finding suggests that just as the practices relevant to resilience need to evolve, so too must the theory and conceptual foundations.

 The gap between theory and practice may indicate a need to further develop the concept of anticipation as it pertains to resilient performance. The concept of anticipation contains what one could call “productive” elements (ie, practices that produce a model of the future) and “limiting” elements (ie, reminders of the limits to our ability to foresee the future). While the limiting elements may be more important to the overall goal of resilient performance, the productive elements may understandably become the focus of efforts to operationalize resilience and anticipation. Explorations of how to manage the tension between these productive and limiting elements may provide an innovative direction for future research regarding RiH.

 An emerging conceptual development in resilience (arising from the fields of ecology^86^ and capacity management,^87^ and elaborated in a recent preprint by Woods^88^) that may help to address this tension is hedging. Hedging is the process by which we maintain or create opportunities to change course after deciding to act in order to protect against adverse outcomes from those decisions.^87,88^ Though rarely mentioned in the literature on RiH, hedging is vital when one must act amidst uncertainty. When facing such circumstances, we must act because adverse events can arise quickly, and we must hedge because our anticipation is imperfect.^88^

 One can make a case for the importance of hedging with the examples of dubious communication. The early statements from NSW Health (that COVID-19 does not spread easily between persons and that face masks are only recommended for individuals who are unwell) and their later reversal reflect the uncertainty that surrounds scientific data when novel circumstances are evolving. When these circumstances present major public health risks (eg, early in a pandemic), there exists pressure to take action and issue guidance amidst that uncertainty. To act in these situations requires anticipation, ie, a model of the future that guides decisions. It also requires maintaining the potential to change course if the chosen action or guidance proves counterproductive (as it did in this case).^88^ While the data collected in this study does not provide insight into how the NSW government avoided anchoring on their original positions (which could have led to significant consequences if maintained), investigating the mechanisms by which governments and other policy-makers hedge represents an intriguing avenue for future theoretical work and empirical research in resilience. Ultimately, incorporating the concept of hedging into healthcare frameworks of resilience could enable a theoretical description of anticipation that is more accessible to practitioners.

###  Limitations

 This study has limitations. Though we attempted to foreground the actions of the governments in both jurisdictions, there remain unknown confounders that influence the capacity for resilient performance in healthcare. Therefore, these findings should not be used to make definitive comparisons between jurisdictions. Instead, they are meant to inform future efforts to develop policies and practices that can enhance the capacity for resilient performance and future research on RiH.

 The use of government-issued media releases yielded a large volume of data for analysis. However, these media releases do not provide a complete picture of the pandemic response. The releases lack information on government deliberations and differences in opinion and worldview amongst individuals. Exploratory issues and issues subject to debate are likely to be resolved internally with only the agreed position released to the public. The result is a succinct release that presents the government as a more uniform entity than is the case.

 Furthermore, these media releases do not serve a singular purpose of disseminating information to the public. These communications serve a social purpose with goals of informing the public, inspiring confidence, and conveying clear public health messages throughout the community. This social purpose may affect communications relevant to the resilience potentials. For example, lessons learned during the pandemic may be processed privately rather than publicly if they cast the government in a negative light.

 Given the potential political implications, a government may have little incentive to communicate its deficiencies to the public. As a result, their media releases may be more likely to contain positive and aspirational content that minimizes the problems in their efforts. Contemporaneous media reports illustrated this potential bias by raising questions regarding claims about enhanced testing capacity,^89,90^ the sufficiency of efforts to increase intensive care capacity,^91-93^ and the commitment to learning from events.^94^ Despite the government’s claims, the media in Ontario was critical throughout the pandemic of what they perceived as the government’s inconsistent application of modelling data and recommendations provided by its scientific advisors.^95^

 These discrepancies reveal a gap between the rhetoric of government media releases and concrete actions at the frontlines. Attention to this gap will need to be an element of future research and efforts to foster resilient healthcare through governmental actions. As suggested by the direct references to resilience, rhetoric that invokes the principles of resilience may be politically beneficial. However, some principles of resilience are likely to meet resistance during their translation into practice (eg, building slack^26,27^ into systems may be opposed as inefficient and fiscally irresponsible) and reduce a government’s commitment to carrying through with their purported priorities. Further exploration of these barriers will benefit from additional methods (eg, direct observation, interviews).

 Notwithstanding these issues, indirect sources such as media releases are frequently used in investigations of resilience,^15^ and the releases we identified contained a wealth of information regarding how these governments acted during the uncertain period of the early COVID-19 pandemic. Though imperfect, they convey important aspects of these governments’ priorities, beliefs, and worldview. Therefore, they can provide useful insights into the actions of governments during disturbances such as COVID-19. While acknowledging its limitations, we believe this study addresses a gap in the literature on RiH, adds to our understanding of how government action can affect resilience, and can inform future research on this issue.

## Conclusion

 This study provides a picture of the relationship between contemporary government actions and the capacity for resilient performance in healthcare. Though governments may not explicitly or consciously focus on resilience, their actions address the resilience potentials. Acknowledging resilience as an important property of systems and communicating a thoughtfully constructed definition of resilience could provide a valuable organizing principle across multiple levels of a system. This effort could also provide an opportunity to foster a greater discourse surrounding the proactive aspects of resilience.

 Our evaluation suggests that, due to the close ties between resilience and the discipline of complexity, the exploration of resilience provides opportunities to re-evaluate assumptions and enhance our understanding of complex socio-technical systems and the unique challenges they present. Capitalizing on these opportunities for double-loop learning could help governments recognize the value of the concept of resilience and prepare for future unexpected events.

 The significance of the gaps between the theoretical foundations of resilience and practical efforts to foster resilience is an issue deserving of increased attention. The resilience potential of anticipation as described theoretically may prove elusive to practitioners seeking resilient performance. While the concrete practices pertaining to resilient performance need further development, the conceptual foundations of the field must also continue to evolve based on the challenges encountered by practitioners. The response to these challenges may determine the ultimate impact of resilience on governments and healthcare systems around the world.

## Ethical issues

 An ethics review was not required as this study used only publicly available data.

## Competing interests

 Authors declare that they have no competing interests.

## Authors’ contributions

 All authors contributed to the conception and design of the study. AS collected the data and conducted much of the data analysis. All authors subsequently contributed to the analysis and interpretation of data. AS drafted the initial manuscript, with all authors contributing to the critical revision and editing of the manuscript for intellectual content. All authors approved the final manuscript as submitted and agree to be accountable for all aspects of the work.

## Supplementary files


Supplementary file 1. Descriptions of the Resilience Potentials and Conceptions of Resilience Used in the Conceptual Framework.

Supplementary file 2. Trends in COVID-19 Cases and Deaths Due to COVID-19 in NSW and Ontario During the Study Period.

Supplementary file 3. Organization of the Governments of NSW and Ontario.

Supplementary file 4. Number of Media Releases Issued by Month During the Study Period.

Supplementary file 5. Additional Quotes of Relevance From Media Releases Issued by the NSW Government and the Government of Ontario During the COVID-19 Pandemic.
