# Supplementary material for: Government Actions and Their Relation to Resilience in Healthcare During the COVID-19 Pandemic in New South Wales, Australia and Ontario, Canada
Source: Int J Health Policy Manag. 2021 Jul 6;11(9):1682–94. doi: 10.34172/ijhpm.2021.67 (PMC9808212; doi:10.34172/ijhpm.2021.67)
Supplement: Supplementary file 1 — Descriptions of the Resilience Potentials and Conceptions of Resilience Used in the Conceptual Framework. [file ijhpm-11-1682-s001.pdf]

Article title: Government Actions and Their Relation to Resilience in Healthcare During the COVID-19 Pandemic in New South Wales, Australia and Ontario, Canada

Journal name: International Journal of Health Policy and Management (IJHPM)

Authors' information: Andrew Smaggus[1]*, Janet C. Long[2], Louise A. Ellis[2], Robyn Clay-Williams[2], Jeffrey Braithwaite[2]

[1]Queen's University, Kingston, ON, Canada.

[2]Australian Institute of Health Innovation, Macquarie University, Sydney, NSW, Australia.

(*Corresponding author: 71acs@queensu.ca)

Supplementary file 1. Descriptions of the Resilience Potentials and Conceptions of Resilience Used in the Conceptual Framework

Resilience Potentials – Fundamental requirements for resilient performance

Potential	Description
Potential to respond[17]	The actions taken following anticipated or unanticipated changes, disturbances, or opportunities
Potential to monitor[17]	Identifying events that signal the need to respond
Potential to anticipate[17]	The capacity to create a model of the future that includes possible disruptions, demands, and opportunities (Anticipating differs from monitoring as monitoring involves observation and anticipating involves imagination)
Potential to learn[17]	The development of new knowledge, skills, and capabilities based on experience

Conceptions of Resilience – Perspectives that guide the pursuit of resilience (ie, the conception of resilience one uses guides selection of the actions that constitute the resilience potentials and the relative amount of attention afforded to each individual potential)

Conception	Description	Notes
Resilience as rebound[3]	Resilience is a system's ability to recover from a disruption	Can lead to a reactive approach to resilience
Resilience as robustness[3]	Resilience is a system's capacity to absorb the effects of a disruption	Can lead to a reactive approach to resilience
Resilience as graceful extensibility[3]	Resilience is a system's ability to expand its capacity in the face of a disruption	Promotes a proactive approach to resilience with greater attention on learning how systems stretch their capacity during an event (rather than how they recover after an event)
Resilience as sustained adaptability[3]	Resilience is a system's ability to adapt to face new challenges and disruptions. This ability is embedded in the structure, organization, and principles of the system	Promotes a proactive approach to resilience with greater attention building infrastructure and principles that support adaptability over the long-term
