# Supplementary material for: Government Actions and Their Relation to Resilience in Healthcare During the COVID-19 Pandemic in New South Wales, Australia and Ontario, Canada
Source: Int J Health Policy Manag. 2021 Jul 6;11(9):1682–94. doi: 10.34172/ijhpm.2021.67 (PMC9808212; doi:10.34172/ijhpm.2021.67)
Supplement: Supplementary file 2 — Trends in COVID-19 Cases and Deaths Due to COVID-19 in NSW and Ontario During the Study Period. [file ijhpm-11-1682-s002.pdf]

Article title: Government Actions and Their Relation to Resilience in Healthcare During the COVID-19 Pandemic in New South Wales, Australia and Ontario, Canada

Journal name: International Journal of Health Policy and Management (IJHPM)

Authors' information: Andrew Smaggus[1]*, Janet C. Long[2], Louise A. Ellis[2], Robyn Clay-Williams[2], Jeffrey Braithwaite[2]

[1]Queen's University, Kingston, ON, Canada.

[2]Australian Institute of Health Innovation, Macquarie University, Sydney, NSW, Australia.

(*Corresponding author: 71acs@queensu.ca)

Supplementary file 2. Trends in COVID-19 Cases and Deaths Due to COVID-19 in NSW and Ontario During the Study Period

a) Cumulative number of cases of COVID-19 reported in New South Wales and Ontario from December 1, 2019 to August 31, 2020

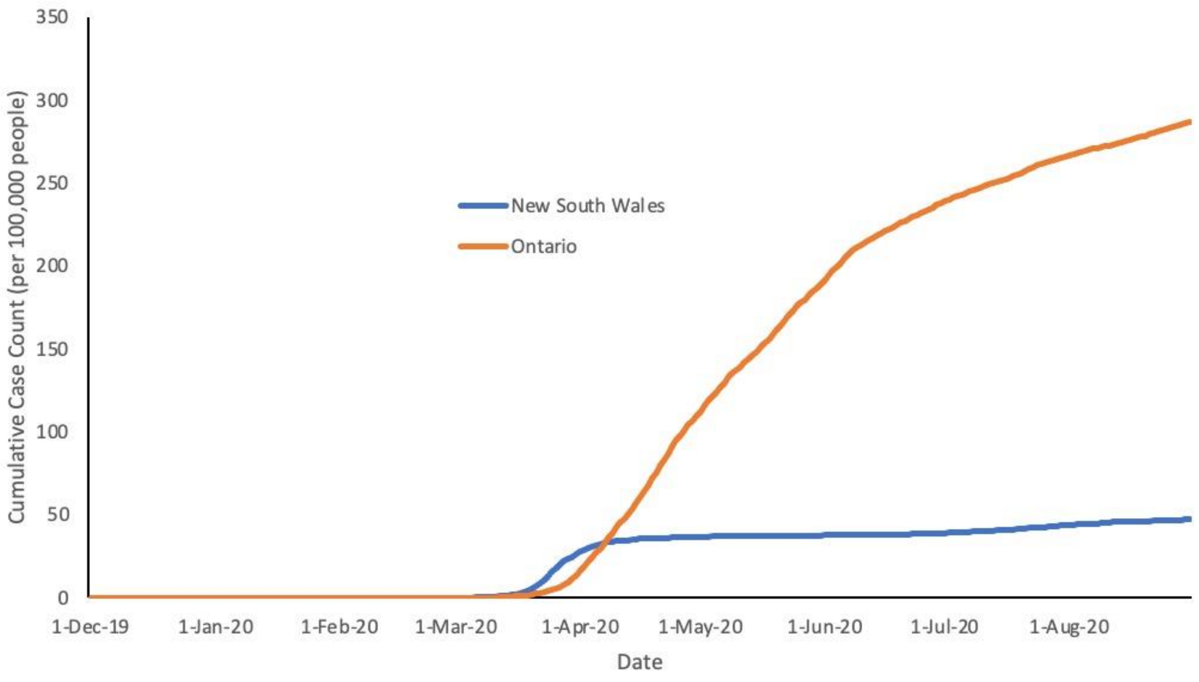

Note: Data obtained from COVID-19 data released by the New South Wales Government,[71] New South Wales Health,[72] and the Government of Ontario.[67] Per capita calculations performed using population data from the Australian Bureau of Statistics[56] and Statistics Canada.[57]

b) Cumulative number of cases of COVID-19 reported in New South Wales and Ontario from December 1, 2019 to August 31, 2020

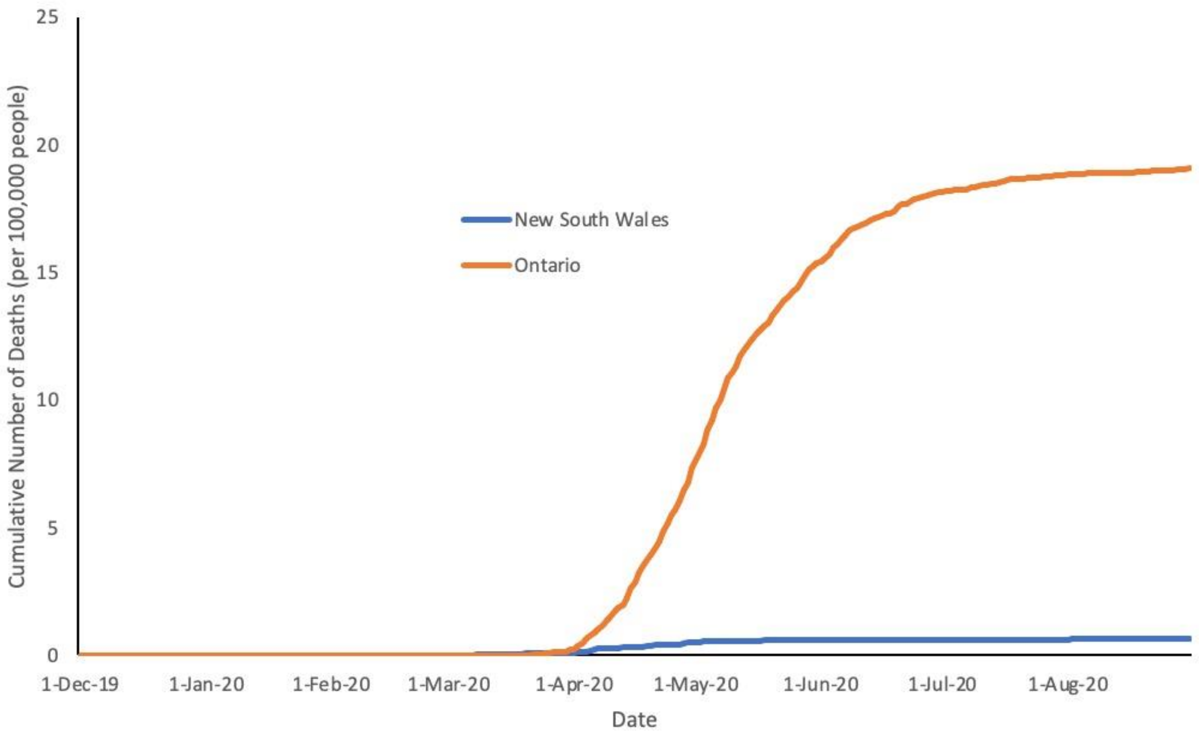

Note: Data obtained from COVID-19 data released by the New South Wales Government,[71] New South Wales Health,[72] and the Government of Ontario.[67] Per capita calculations performed using population data from the Australian Bureau of Statistics[56] and Statistics Canada.[57]
