# Supplementary material for: Government Actions and Their Relation to Resilience in Healthcare During the COVID-19 Pandemic in New South Wales, Australia and Ontario, Canada
Source: Int J Health Policy Manag. 2021 Jul 6;11(9):1682–94. doi: 10.34172/ijhpm.2021.67 (PMC9808212; doi:10.34172/ijhpm.2021.67)
Supplement: Supplementary file 3 — Organization of the Governments of NSW and Ontario. [file ijhpm-11-1682-s003.pdf]

Article title: Government Actions and Their Relation to Resilience in Healthcare During the COVID-19 Pandemic in New South Wales, Australia and Ontario, Canada

Journal name: International Journal of Health Policy and Management (IJHPM)

Authors' information: Andrew Smaggus[1]*, Janet C. Long[2], Louise A. Ellis[2], Robyn Clay-Williams[2], Jeffrey Braithwaite[2]

[1]Queen's University, Kingston, ON, Canada.

[2]Australian Institute of Health Innovation, Macquarie University, Sydney, NSW, Australia.

(*Corresponding author: 71acs@queensu.ca)

Supplementary file 3. Organization of the Governments of NSW and Ontario

a) Departments and ministries of the New South Wales Government

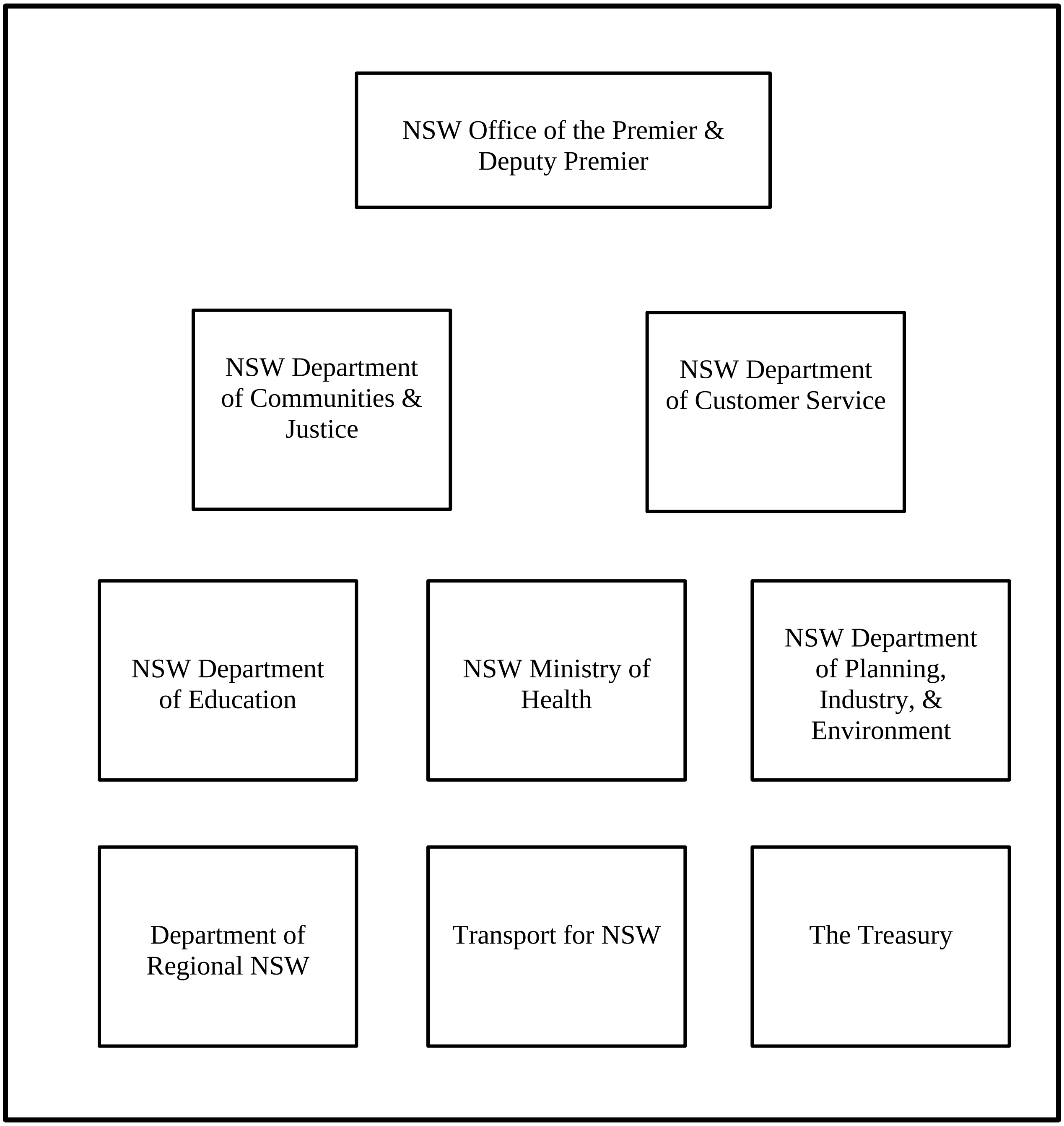

Abbreviation: NSW, New South Wales.

b) Departments and ministries of the Government of Ontario

Office of the Premier

Ministry of Agriculture, Food, & Rural Affairs	Ministry of the Attorney General	Ministry of Children, Community, & Social Services
Ministry of Colleges & Universities	Ministry of Economic Development, Job Creation, & Trade	Ministry of Education
Ministry of Energy, Northern Development, & Mines	Ministry of the Environment, Conservation, & Parks	Ministry of Finance
Ministry of Francophone Affairs	Ministry of Government & Consumer Services	Ministry of Health
Ministry of Heritage, Sport, Tourism, & Culture Industries	Ministry of Indigenous Affairs	Ministry of Infrastructure
Ministry of Intergovernmental Affairs	Ministry of Labour, Training, & Skills Development	Ministry of Long-Term Care
Ministry of Municipal Affairs & Housing	Ministry of Natural Resources & Forestry	Ministry for Seniors & Accessibility
Ministry of the Solicitor General	Ministry of Transportation	Treasury Board Secretariat
