# Supplementary material for: Government Actions and Their Relation to Resilience in Healthcare During the COVID-19 Pandemic in New South Wales, Australia and Ontario, Canada
Source: Int J Health Policy Manag. 2021 Jul 6;11(9):1682–94. doi: 10.34172/ijhpm.2021.67 (PMC9808212; doi:10.34172/ijhpm.2021.67)

Article title: Government Actions and Their Relation to Resilience in Healthcare During the COVID-19 Pandemic in New South Wales, Australia and Ontario, Canada

Journal name: International Journal of Health Policy and Management (IJHPM)

Authors' information: Andrew Smaggus[1]*, Janet C. Long[2], Louise A. Ellis[2], Robyn Clay-Williams[2], Jeffrey Braithwaite[2]

[1]Queen's University, Kingston, ON, Canada.

[2]Australian Institute of Health Innovation, Macquarie University, Sydney, NSW, Australia.

(*Corresponding author: 71acs@queensu.ca)

Supplementary file 4. Number of Media Releases Issued by Month During the Study Period

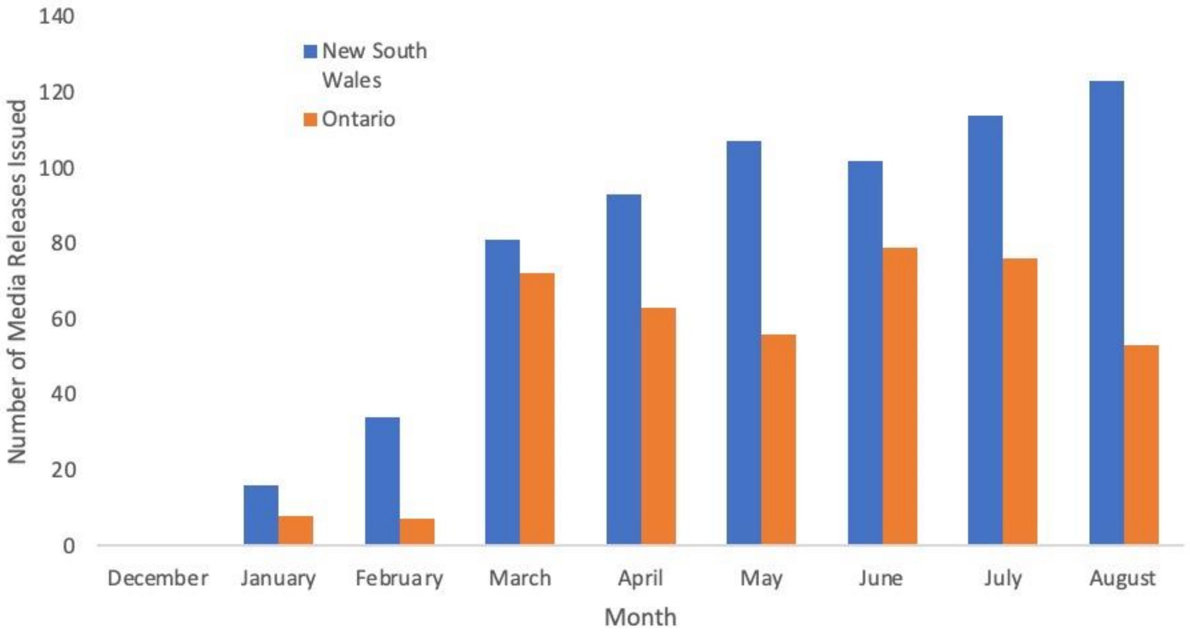

Supplement: Supplementary file 4 — Number of Media Releases Issued by Month During the Study Period. [file ijhpm-11-1682-s004.pdf]
