# Supplementary material for: Government Actions and Their Relation to Resilience in Healthcare During the COVID-19 Pandemic in New South Wales, Australia and Ontario, Canada
Source: Int J Health Policy Manag. 2021 Jul 6;11(9):1682–94. doi: 10.34172/ijhpm.2021.67 (PMC9808212; doi:10.34172/ijhpm.2021.67)
Supplement: Supplementary file 5 — Additional Quotes of Relevance From Media Releases Issued by the NSW Government and the Government of Ontario During the COVID-19 Pandemic. [file ijhpm-11-1682-s005.pdf]

**Article title:** Government Actions and Their Relation to Resilience in Healthcare During the COVID-19 Pandemic in New South Wales, Australia and Ontario, Canada

**Journal name:** International Journal of Health Policy and Management (IJHPM)

**Authors' information**: Andrew Smaggus[1]*, Janet C. Long[2], Louise A. Ellis[2], Robyn Clay-Williams[2], Jeffrey Braithwaite[2]

[1]Queen's University, Kingston, ON, Canada.

[2]Australian Institute of Health Innovation, Macquarie University, Sydney, NSW, Australia.

(*Corresponding author: 71acs@queensu.ca)

**Supplementary file 5.** Additional Quotes of Relevance From Media Releases Issued by the NSW Government and the Government of Ontario During the COVID-19 Pandemic

| **Additional Quotes** |
| --- |
| Previous Experiences with SARS |
| *Redeploying inspectors to help with critical areas of need was also done in the long-term care sector during SARS in 2003. This model was effective in supporting homes through that pandemic.* (Ontario News Release 2020 03 28)<br><br>*In 2003, the Ontario government appointed an independent commission to investigate the introduction and spread of Severe Acute Respiratory Syndrome (SARS) under the Health Protection and Promotion Act. The commission interviewed 600 people and held six days of public hearings.* (Ontario News Release 2020 07 29)<br><br>*NSW Health has developed and exercised a range of procedures for case finding, diagnosis, and contact tracing for high consequence infectious diseases (such as pandemic influenza, SARS, MERS, and emerging infections) should they occur in NSW.* (NSW Health Release 2020 01 21)<br><br>*The NSW Health response to COVID-19 is part of an existing framework for managing emerging infectious diseases, including pandemic influenza, SARS and MERS in the past, which has been refined over many years.* (NSW Health Release 2020 02 13) |

Abbreviations: NSW, New South Wales; SARS, Severe Acquired Respiratory Syndrome; MERS, Middle East Respiratory Syndrome.
